# Effect of Hyperbaric Oxygen Therapy on Acute Liver Injury and Survival in a Rat Cecal Slurry Peritonitis Model

**DOI:** 10.3390/life10110283

**Published:** 2020-11-15

**Authors:** Hee Won Yang, Sangchun Choi, Hakyoon Song, Min Ji Lee, Ji Eun Kwon, Han A. Reum Lee, Kyuseok Kim

**Affiliations:** 1 Department of Emergency Medicine, Ajou University School of Medicine, Suwon 16499, Korea; speedheewon@gmail.com (H.W.Y.); hakyoon.song@gmail.com (H.S.); 2 Department of Emergency Medicine, Seoul National University College of Medicine, Seoul 03080, Korea; minji.lee29@gmail.com; 3 Department of Pathology, Ajou University School of Medicine, Suwon 16499, Korea; kjefullup@aumc.ac.kr; 4 Department of Emergency Medicine, CHA University School of Medicine, Seongnam 13497, Korea; harlee91@naver.com

**Keywords:** hyperbaric oxygenation, sepsis, microcirculation, apoptosis, survival rate

## Abstract

Background: The effects of hyperbaric oxygen therapy (HBOT) in sepsis remain unclear. This study evaluated its effects on acute liver injury and survival in a rat model. Methods: Cecal slurry peritonitis was induced in male rats, which were then randomly allocated into the HBOT and control groups. In the survival experiment, six 90 min HBOT sessions (2.6 atmospheres absolute 100% oxygen) were performed over 48 h; the survival rate was determined 14 days after sepsis induction. In the acute liver injury experiment, three HBOT sessions were performed, followed by liver and plasma harvesting, 24 h after sepsis induction. Serum levels of alanine aminotransferase (ALT), interleukin (IL)-6, and IL-10 were measured, and the hepatic injury scores were determined. Reactive oxygen species (ROS) generation was detected by 2′,7′-dihydrodichlorofluorescein diacetate (H2DCF-DA) assay. Western blot assays assessed protein kinase B (Akt), phosphorylated-Akt (p-Akt), glycogen synthase kinase (GSK)-3β, phosphorylated-GSK-3β, and cleaved caspase-3 levels. Results: Survival in the HBOT group (57.1%) was significantly higher than that in the controls (12.5%, *p* = 0.029), whereas IL-6, IL-10, and ALT levels were significantly lower in the HBOT group. The ROS generation was significantly inhibited to a greater extent in the HBOT group than in the control group. Additionally, in the HBOT group, the p-Akt and p-GSK-3β increased significantly and cleaved caspase-3 levels decreased significantly. Conclusions: HBOT showed a beneficial effect on acute liver injury and rat survival by enhancing the Akt signaling pathway and decreasing apoptosis.

## 1. Introduction

Sepsis is a life-threatening, inflammatory organ dysfunction that affects millions of people, worldwide [1,2]. Surviving sepsis requires urgent assessment and treatment, including early recognition of the disease, fluid resuscitation, broad-spectrum antibiotic therapy, and on-going patient re-evaluation [3]. Because our understanding of its pathophysiology is complicated and not sufficiently evolved, at present, the sepsis treatment guideline emphasizes a timely, conservative care protocol; a specific treatment has not been developed.

A critical process that is up-regulated in patients experiencing septic shock is systemic inflammation. During the sustained hyper-metabolic period, systemic inflammation causes depletion of cellular glutathione and can lead to tissue accumulation of toxic oxidizing agents. The presence of highly toxic oxidizing moieties in the bloodstream can cause systemic microangiopathic dysfunction, resulting in multiple organ injuries. Systemic microvascular injury is considered to play a key role in the pathogenesis of sepsis as it significantly increases endothelial permeability [4,5]. Therefore, treatments that improve systemic microangiopathic dysfunction are needed to effectively treat this disease.

Hyperbaric oxygen therapy (HBOT) may have beneficial effects on the treatment of sepsis [6,7,8,9,10]. These effects are presumed to be mediated by the increased level of dissolved oxygen in the blood, which results in an oxygen partial pressure of approximately 2200 mmHg and a blood oxygen concentration of approximately 27 mL/dL at 3 Atmospheres absolute (ATA) [11,12], increasing the oxygen available to cells. HBOT can also stimulate both vasoconstrictive responses and angiogenesis [13]. Furthermore, hyperbaric hyperoxia enhances the antimicrobial activity of immune cells by increasing their respiratory burst and phagocytic activities [14]. However, few experimental studies have investigated the effects of HBOT on sepsis. One of the HBOT responses that may benefit patients suffering from septic shock includes an enhanced antioxidant response [13]. For example, in human endothelial cells, HBO exposure upregulates the expression of antioxidant genes that provide resistance to reactive oxygen species [15]. Furthermore, an earlier study revealed that HBOT increases the efficacies of several antibiotics via an oxygen-dependent transport system [16]. Consequently, HBOT may improve systemic microcirculation and induce anti-apoptotic effects in patients with sepsis. This study evaluated the effects of HBOT on survival and acute liver injury in a rat model of severe sepsis.

## 2. Materials and Methods

### 2.1. Animals

Ajou University Medical Center, Institutional Animal Care and Use Committee approved the experimental procedures used in the present study (AUMC-IACUC-2018-0024). All methods regarding experiments were carried out in accordance with relevant guidelines and regulations. A total of 44 male Sprague–Dawley rats, weighing 300–350 g, were used in this study. The animals were housed under strictly controlled conditions of light and temperature, and were provided with standard rations and water ad libitum for at least 7 days prior to the experiment.

### 2.2. Polymicrobial Sepsis Model

Polymicrobial peritoneal sepsis was induced in the rats using a modified cecal slurry procedure [17]. Eight donor rats were anesthetized using isoflurane, followed by intramuscular injection of a tiletamine and zolazepam combination (50 mg/kg) and xylazine (10 mg/kg). Thereafter, a laparotomy was performed along the midline of each animal and the cecum was extruded. A 0.5 cm incision was made in the anti-mesenteric surface of the cecum, and the contents were manipulated to expel fecal material. The fecal material from each animal was collected and weighed. Immediately after collection, the fecal materials from different donor animals were diluted 1:3 with a 5% dextrose solution and mixed to create a homogeneous suspension. The suspension of fecal material from the donors was intraperitoneally administered (5 mL/kg) to each of the 36 experimental rats. New cecal slurries were prepared from donor rats prior to each experiment and used within 2 h of preparation. After closing the abdomen, each animal received an intramuscular injection of ceftriaxone (150 mg/kg), and additional 5% dextrose solution (30 mL/kg) was subcutaneously administered for fluid resuscitation.

### 2.3. Hyperbaric Oxygen Therapy (HBOT)

Rats were randomly assigned to the HBOT and control groups. HBOT was administered using a small rodent HBO chamber (IBEX Medical Systems, Wonju, Korea). Animals undergoing HBOT were placed in the HBO chamber, which was then pressurized using 100% oxygen compression (5 psi/min). Based on previous experiments, HBOT was administered at 2.6 ATA [8]. Similarly, decompression also occurred at a rate of 5 psi/min. HBOT sessions (90 min, each) were administered every 8 h for 24 h. The last HBOT session was performed 2 h before the animals were euthanized. The control group was similarly exposed to normobaric conditions in a similar chamber to control for the effects of hypothermia and experimental stress. The HBOT and control animals each received a daily intramuscular injection of ceftriaxone (50 mg/kg).

### 2.4. Experimental Protocols

In the first experiment, examining survival of the animals in the HBOT (n = 8) and control groups (n = 8), a total of six HBOT sessions were performed over a 48 h period. Thereafter, the animals were observed for a period of 14 days from the time of sepsis induction.

In the second experiment, blood and tissues were harvested 24 h after sepsis induction to measure indicators of organ injuries in the HBOT group (n = 8) and the control group (n = 8), and a total of three HBOT sessions were performed over a 24 h period. In this experiment, the animals were anesthetized using the same injectable anesthetics as those used prior to collection of the fecal material. Then, a midline laparotomy was performed, and the animals were exsanguinated by collecting blood from the abdominal aorta into a heparinized syringe. The blood pH; oxygen partial pressure; carbon dioxide partial pressure; base excess; and levels of interleukin (IL)-6, IL-10, glucose, alanine transaminase (ALT), reactive oxygen species (ROS) generation, manganese superoxide dismutase (SOD), and lactate were measured. Liver tissues were also harvested and stored at −70 °C for subsequent analyses. Protein kinase B (Akt), phosphorylated-Akt (p-Akt), glycogen synthase kinase (GSK)-3β, phospho-GSK-3β (p-GSK-3β), and cleaved caspase-3 levels were determined using Western blot assays.

### 2.5. Laboratory Analyses

Arterial blood gas analyses were performed immediately after collection of the blood samples. The remaining blood was centrifuged (15 min at 3000 rpm, 4 °C); the separated plasma was collected and stored at −70 °C until needed for the blood chemistry profile analyses. Serum lactic acid, albumin, amylase, alanine aminotransferase (ALT), and glucose levels were measured using a blood gas analyzer (RAPIDPoint^®^500 Blood Gas Systems, Siemens Healthcare Diagnostics, Munich, Germany) and a chemistry analyzer (VetScan VS2, Abaxis, Union City, CA, USA).

### 2.6. Cytokine Analyses

Enzyme-linked immunosorbent assays were performed, according the manufacturer’s specifications (R&D Systems, Minneapolis, MN, USA), to quantify the IL-6 and IL-10 levels.

### 2.7. Quantitative Analyses of Intracellular ROS Generation and Maganase Superoxide

Intracellular ROS generation of hepatocytes was measured using a 2′,7′-dihydrodichlorofluorescin diacetate (H2DCF-DA) assay (Sigma, St. Louis, MO, USA). Frozen liver tissues were weighted, thawed, homogenized in the 500 µL ice-cold radioimmunoprecipitation assay (RIPA) buffer, and then centrifuged at 2500 rpm, at 4 °C for 10 min. The supernatant after centrifugation was added to the reaction mixture containing H2DCF-DA (10 µM/mL) and incubated at 37 °C for 60 min. Fluorescence intensity was measured using a fluorescence plate reader with an excitation wavelength of 480 nm and an emission wavelength of 530 nm, and its values were presented in fluorescence intensity per milligram of tissue.

The level of manganese superoxide dismutase (MnSOD) was measured to examine the cellular antioxidant capacity using the MnSOD ELISA assay kit (R&D Systems, Minneapolis, MN, USA).

### 2.8. Western Blot Anlaysis

To investigate the survival pathway in liver tissue of a polymicrobial septic rat model, Western blot analysis was performed for liver tissue of a polymicrobial septic rat model, and the level of Akt, phosphorylated Akt, GSK-3β, phosphorylated GSK-3β, and caspase-3 using liver tissue was evaluated. We procured primary antibodies against total Akt (Cat. No. 4691, RRID:AB_915783), phospho-Akt (Ser473) (Cat. No. 4058, RRID:AB_331168), total GSK-3β (Cat. No. 9315, RRID:AB_490890), phospho-GSK-3β (Ser9) (Cat. No. 9323, RRID:AB_2115201), caspase-3 (Cat. No. 9662, RRID:AB_331439), cleaved caspase-3 (Cat. No. 9664, RRID:AB_2070042) (Cell Signaling Technology, Danvers, MA, USA), and primary antibodies against β-actin (Cat. No. sc-47778, RRID:AB_2714189) (Santa Cruz Biotechnology Inc., Santa Cruz, CA, USA). Goat anti-rabbit or anti-mouse horseradish peroxidase (HRP)-conjugated secondary antibodies were also provided (Promega, Madison, WI, USA). Liver tissues were homogenized in radio-immunoprecipitation assay (RIPA) lysis buffer containing protease and phosphatase inhibitor cocktail (Thermo Fisher Scientific, Waltham, MA, USA) on ice. The protein extracts were quantified by the bicinchoninic acid (BCA) protein assay kit (Thermo Fisher Scientific, Waltham, MA, USA). We separated proteins (30 μg) by 12% SDS-PAGE (SDS-polyacrylamide gel electrophoresis) and followed by an electrophoretic transfer onto a polyvinylidene difluoride (PVDF) membrane (GE Healthcare, Chicago, IL, USA) in transfer buffer (25 mM Tris-base, 192 mM glycine, 20% methanol). The blots were blocked in TBS-T (Tris-buffered saline with 0.05% Tween 20) (Bio-Rad, Hercules, CA, USA) containing 5% nonfat dry milk (Difco, Detroit, MI, USA) to prevent non-specific antibody binding, and then incubated with 1000-fold diluted rabbit antibodies against GSK-3β, phospho-GSK-3β, caspase-3 at 4 °C overnight. The membranes were washed and further incubated with 2000-fold diluted HRP-conjugated secondary antibodies at room temperature for 1 h. The bands were photographed using ImageQuant LAS 4000 (GE Healthcare, Chicago, IL, USA) with Clarity Max Western Enhanced chemiluminescence (ECL) Substrate (Bio-Rad, Hercules, CA, USA), and the intensity of the bands was quantified using image J analysis software (NIH).

### 2.9. Histological Hepatic Injury Severity Score

Liver tissue was fixed in 4% formalin and embedded in paraffin. Each paraffin block was sectioned (4 µm thick) and stained with hematoxylin and eosin. Hepatic injuries were then scored by one pathologist, blinded to the treatment group. The hepatic injuries were assessed using morphologic criteria: spotty necrosis (range, 0–4), capsular inflammation (range, 0–3), portal capsular inflammation (range, 0–3), ballooning degeneration capsular inflammation (range, 0–3), and steatosis capsular inflammation (range, 0–3). These aggregated scores were used to determine a hepatic injury severity score, ranging from 0 (none) to 16 (severe).

### 2.10. Statistical Analysis

Those variables that did not follow a normal distribution are reported as medians, percentiles, and quartiles. Categorical variables are expressed as frequencies and percentiles. Continuous data are reported as medians (interquartile range, IQR) and were compared using the Mann–Whitney *U* test. The mortality rate was calculated and illustrated using Kaplan–Meier curves. Differences with *p*-values < 0.05 were considered statistically significant; all statistical analyses were performed using SPSS, version 24.0 (SPSS, Chicago, IL, USA)

## 3. Results

### 3.1. Survival Rate

**Figure 1:**
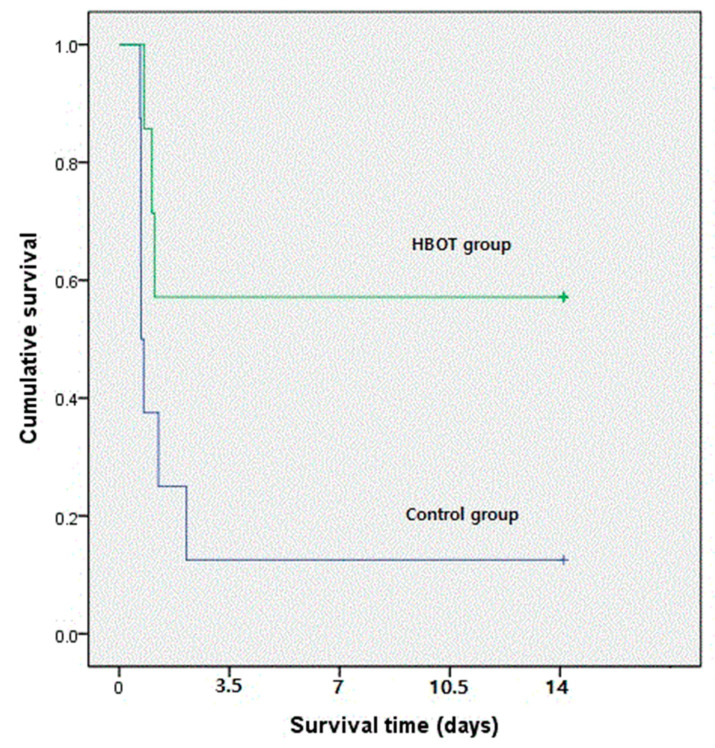
Rat survival following induction of peritoneal sepsis and treatment with hyperbaric oxygen therapy (green line) versus controls (blue line). Survival rate at 14 days, hyperbaric oxygen therapy (HBOT) group—57.1%; control group—12.5%. (*p* = 0.029, Kaplan–Meier analysis).

The survival rate, at 14 days, in the HBOT group (n = 8) was significantly higher than that in the control group (n = 8) (57.1% vs. 12.5%, *p* = 0.029) (Figure 1).

### 3.2. Laboratory Analyses

Serum lactic acid, albumin, amylase, ALT, and glucose levels are summarized in Table 1. In the HBOT group, the ALT and glucose levels were significantly better than those in the control group (*p* = 0.02). Serum albumin and amylase levels were not affected by HBOT, but the lactate and base excess levels were significantly lower than those in the control group (*p* < 0.05).

### 3.3. Sepsis-Related Cytokines

The IL-6 and IL-10 levels are shown in Table 2, along with the IL-6/IL-10 ratios. In the HBOT group, the IL-6 and IL-10 levels were significantly lower than in the control group (both, *p* < 0.05). The IL-6/IL-10 ratio was also significantly lower in the HBOT group (*p* = 0.04).

### 3.4. Intracellular ROS Generation and Manganese SOD

**Figure 2:**
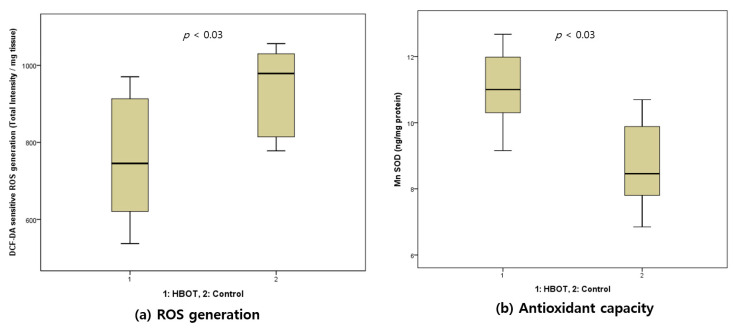
Dihydrodichlorofluorescin diacetate (DCF-DA) assay and manganese superoxide dismutase (MnSOD) assay results in rats with induced peritoneal sepsis and treated with hyperbaric oxygen therapy (HBOT) versus controls. The intracellular ROS level in hepatic tissues (**a**) decreased significantly in the HBOT group more than in the control group (*p* < 0.03). In addition, the increase in antioxidant activity (**b**) was more significant in the HBOT group than in the control group (*p* < 0.03).

The DCF-DA level in hepatic tissues decreased more in the HBOT group (745.4 (600.0–927.5)) than in the control group (978.7 (805.2–1036.6)) (*p* < 0.03). On the contrary, the MnSOD level in hepatic tissues increased more in the HBOT group (11.0 (10.3–12.2)) than in the control group (8.5 (7.6–10.3)) (*p* < 0.03) (Figure 2).

### 3.5. Western Blot Analysis

**Figure 3:**
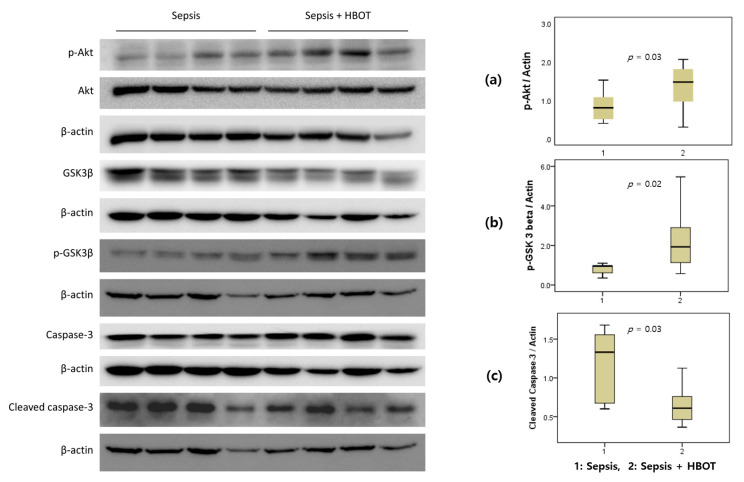
Western blot analysis of results of phosphorylated protein kinase B (p-Akt), phosphorylated glycogen synthase kinase (p-GSK-3β), and cleaved caspase-3 in rats with induced peritoneal sepsis and treated with hyperbaric oxygen therapy (HBOT) versus controls. The p-Akt (**a**) and p-GSK-3β (**b**) significantly increased in the HBOT group (*p* < 0.05 for both), whereas the expression of cleaved caspase-3 (**c**) decreased significantly in the HBOT group (*p* = 0.02).

The p-Akt/actin ratio was significantly higher in the HBOT group (1.48 (0.88–2.07)) than in the control group (0.80 (0.49–1.10), *p* = 0.03). The p-GSK-3β/actin ratio was significantly higher in the HBOT group (1.92 (1.00–3.54)) than in the control group (0.96 (0.43–0.98), *p* = 0.02). However, the cleaved caspase-3/actin ratio was significantly decreased in the HBOT group (0.61 (0.42–0.79)) than in the control group (1.33 (0.67–1.61), *p* = 0.03) (Figure 3).

### 3.6. Histological Hepatic Injury Scores

**Figure 4:**
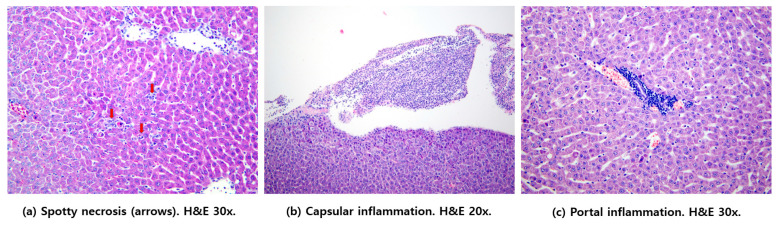
Representative histologic findings in the Control group. (**a**) Several adjacent hepatocytes were replaced by inflammatory cells. (**b**) Moderate amounts of inflammatory cells were infiltrated in the capsule. (**c**) Inflammatory cellular infiltration was evident in the portal area.

The hepatic injury score was lower in the HBOT group (5 (4.25–6.75)) than in the control group (7 (4.0–7.0)). However, the difference was not statistically significant. Spotty necrosis, capsular inflammation and portal inflammation in the Control group are shown in Figure 4.

## 4. Discussion

Novel strategies for the treatment sepsis are needed. Our study showed that the survival rate for rats treated with HBOT was 57.1%, whereas in the control group, the survival rate was only 12.5% (*p* < 0.05) during the 14-day observation period. Although previous research has demonstrated the beneficial effects of HBOT, several differences were evident between our experiments and those in previously published studies [6,7,8,9,10]. First, the point at which previous studies measured survival was shorter (3–4 days), and the HBOT timings and pressures were varied. Second, the control group survival rates reported in several studies were between 50% and 60%, which are too high to translate to survival rates in humans with sepsis. Lastly, several experimental studies reported that HBOT improved survival rates in the cecal ligation and puncture (CLP) sepsis model. Thus, in our opinion, the previous studies involved less severe sepsis models that resulted in the control groups having mortality rates that were too low to accurately reflect the situation in patients with severe sepsis. Further, the polymicrobial model of sepsis in Sprague–Dawley rats that we used has been demonstrated to induce hemodynamic and physiological changes similar to those in humans with sepsis [17].

Our results also suggest that liver injury in rats with sepsis may be attenuated using HBOT. The liver is well-known to be a potent scavenger of blood-borne bacteria and their inflammatory byproducts [18]. Although a previous study demonstrated that HBOT reduced liver injury, it failed to demonstrate any survival improvement relative to the controls [7]. HBOT is known to reduce hepatocellular degeneration and neutrophil infiltration and to reduce the ability of neutrophils to adhere to liver tissue [19]. Remarkably, in the fecal slurry model, serum ALT levels were significantly improved in the HBOT group when compared with the control group; serum ALT is a sensitive and translatable indicator of hepatotoxicity [20]. However, in our study, the histologic hepatic injury scores did not show a significant difference between the two groups. Rather, the score in the HBOT group tended to be lower than in the control animals. One mechanism by which HBOT is suspected to enhance the treatment of severe sepsis is that both apoptosis inhibition and metabolism improvements may occur in individuals subjected to hyperoxia. Thus, these effects might take more time to be reflected in the observed histological changes, reducing the difference in the hepatic injury scores between the experimental groups. Further, since HBOT is a potential adjunctive treatment for severe sepsis, the rats in our model also received antimicrobial chemotherapy and fluid resuscitation during the acute stage of severe sepsis. Since these procedures are the mainstay of sepsis treatment, the adjunctive effect of HBOT may be more difficult to demonstrate in severe disease. In addition, because we used a severe sepsis model, the degree of improvement may have been less than might be observed in a milder model of disease. Ultimately, further study is needed to clarify the effects of HBOT on histologic hepatic injury in severe sepsis.

According to Woźnica et al. [21], the proposed mechanisms of liver injury by sepsis are hyperinflammation, microcirculatory failure, and side effects of the therapy. In our study, high cytokine levels might indicate that liver injury by sepsis occurred by hyperinflammation, which was attenuated by HBOT. In addition, the IL-6 and IL-10 levels as well as the IL-6/IL-10 ratio, which has been known as a surrogate marker of inflammation [22], were significantly lower in the HBOT group than in the controls. Higher concentrations of IL-6 and IL-10 predict higher mortality rates in patients with sepsis [23]; an elevated IL-6/IL-10 ratio is also associated with mortality in these patients [24]. These findings support the survival gains observed in rats with induced sepsis and treated with HBOT in our study.

The intracellular ROS generation in hepatic tissues significantly decreased in the HBOT group more than in the control group. In contrast, the MnSOD level in hepatic tissues in the HBOT group increased more than that for the control group (*p* < 0.03 for both). These results are concordant with the results from the previous studies [13,15], supporting the theory that HBOT’s antioxidant effect partially mitigates its therapeutic effects.

The control of cell signaling pathways is an important treatment option for cytokine regulation in the sepsis model. Cross et al. demonstrated that GSK-3β is phosphorylated at its N-terminus (serine 9 residue) via the Akt pathway [25]. In bone marrow stromal cells, HBOT has been demonstrated to increase GSK-3β phosphorylation through the PI3 kinase-Akt survival pathway [26]. In concordance with the results from the above studies, our study found that pGSK-3β expression in animals was significantly increased by HBOT, but this increase was absent in the control groups. HBOT appears to reduce hyperinflammatory reactions through modifying cell signaling in this sepsis model; however, the anti-apoptotic mechanism involving Akt activation and GSK-3β inhibition needs further investigation.

GSK-3β expression is correlated with the regulation of caspase-3 and -8, both of which can induce the activation of pro-apoptotic proteins, and with the release of cytochrome c, which can be triggered by the mitochondrial-mediated programmed cell death pathway [27]. Caspase-3 can be activated by both the extrinsic and intrinsic apoptosis pathways, and its crucial role in programmed cell death has been demonstrated in a septic mouse model [28]. A previous study revealed that the administration of drugs that block caspase activity results in anti-apoptotic effects and improves the survival rate of mice with sepsis [29]. Moreover, caspase-3 activation and focal apoptosis have been documented in the spleens from 56% of patients with sepsis [30]. In the present study, HBOT showed a similar caspase blocking effect. In the HBOT animals, cleaved caspase-3 levels were significantly decreased compared with the control animals, supporting our contention that HBOT inhibits the apoptotic pathway in this sepsis model. Although the precise mechanism through which HBOT protects against sepsis remains unclear, the regulation of cytokine activity and inflammation may play important roles.

Our study has several limitations. First, we did not evaluate the direct bactericidal effects of HBOT, in our experiments; anaerobic pathogens are reportedly unable to survive under HBO conditions [6,31]. For example, *Clostridium* spp. are unable to survive in an oxygenated environment [13]. According to previous investigations, there is some debate regarding the direct bactericidal effects of HBOT on infectious organisms. A recent study showed that the bacterial load within the peritoneal fluid and blood was not affected by HBOT in the CLP sepsis model. The authors concluded that, in that polymicrobial sepsis model, HBOT did not have a direct bactericidal effect [32]. However, our study results may not be affected by the presence or absence of a direct, HBOT-mediated bactericidal effect. Further study will be needed to clarify the bactericidal effect of HBOT on the survival mechanism. Second, although we found that the levels of cleaved caspase-3 were significantly lower in animals treated with HBOT, we could not directly measure the molecule(s) mediating the antiapoptotic effect or altering mitochondrial function. Further investigation is needed to directly evaluate mitochondrial function, following HBOT, in the sepsis model. Third, the animal hyperbaric chamber that was used is too small to precisely regulate the pressure within the chamber, making barotrauma one of the limitations of the present study. However, as mentioned above, this study employed a previously validated pressure control method. Fourth, AKT/GSK 3 pathways are cell specific [33], and in the liver, there are many different cells, including hepatocytes, Kupffer cells, and endothelial cells. We could not investigate which cells are more responsible for the AKT/GSK 3 pathway findings in this study.

In conclusion, HBOT showed a beneficial effect on acute liver injury and overall animal survival in a cecal slurry model of peritonitis. This effect would be mediated through enhancement of the PI3K/Akt signaling pathway and by decreasing apoptosis. Thus, a future goal may be to tailor therapies to target the GSK3β-mediated pathways that contribute to uncontrolled inflammation, and accelerate organ dysfunction and failure in patients with sepsis.

## Figures and Tables

**Table 1:** Laboratory blood analysis results in rats with induced peritoneal sepsis receiving treatment with hyperbaric oxygen therapy (HBOT) versus controls.

Parameter	HBOT Group	Control Group	*p*-Value
**pH**	7.4 (7.31–7.42)	7.3 (7.2–7.4)	0.25
**PCO_2_ (mmHg)**	35.2 (25.0–37.6)	26.9 (19.4–45.8)	0.74
**PO_2_ (mmHg)**	103.3 (92.2–114.63)	94.3 (58.8–112.3)	0.44
**HCO_3_ (mmol/L)**	18.2 (15.6–22.0)	15.7 (11.4–18.0)	0.10
**Lactate (mmol/L)**	2.8 (2.3–3.9)	5.9 (3.5–6.6)	0.02
**Base excess (mmol/L)**	−6.3 (−9.5 to −3.2)	−10.6 (−14.7 to −7.6)	0.03
**Albumin (g/dL)**	3.8 (3.6–4.0)	3.8 (3.6–3.9)	0.91
**ALT (IU/L)**	34.0 (31.0–38.0)	46.5 (37.3–163.8)	0.02
**Amylase (IU/L)**	730.0 (634.0–1194.0)	976.0 (742.5–1137.0)	0.27
**Glucose (mg/dL)**	121.0 (115.0–137.0)	101.5 (65.3–111.8)	0.04

Data are presented as medians (Interquartile range, IQR). PCO_2_, carbon dioxide partial pressure; PO_2_, oxygen partial pressure; HCO_3_, bicarbonate; ALT, alanine transaminase.

**Table 2:** Serum interleukin (IL) levels in rats with induced peritoneal sepsis receiving hyperbaric oxygen therapy (HBOT) versus controls.

Variable	HBOT Group	Control Group	*p*-Value
**IL-6 (pg/mL)**	4.5 × 10^3^ (3.7 × 10^3^–8.2 × 10^3^)	23 × 10^3^ (88 × 10^3^–298 × 10^3^)	0.01
**IL-10 (pg/mL)**	3.3 × 10^3^ (3.0 × 10^3^–4.2 × 10^3^)	4.3 × 10^3^ (3.6 × 10^3^–6.6 × 10^3^)	0.04
**IL-6/IL-10 ratio**	1.5 (1.1–1.9)	3.9 (1.8–5.6)	0.04

Data are presented as medians (Interquartile range, IQR).

## Data Availability

Data are the property of the authors and can become available by contacting the corresponding author.
